# The effect of wrist posture on extrinsic finger muscle activity during single joint movements

**DOI:** 10.1038/s41598-020-65167-x

**Published:** 2020-05-20

**Authors:** Carl R. Beringer, Misagh Mansouri, Lee E. Fisher, Jennifer L. Collinger, Michael C. Munin, Michael L. Boninger, Robert A. Gaunt

**Affiliations:** 1 Rehab Neural Engineering Labs, University of Pittsburgh, Pittsburgh, PA 15213 USA; 2 Department of Bioengineering, University of Pittsburgh, Pittsburgh, PA 15213 USA; 3 Center for the Neural Basis of Cognition, Pittsburgh, PA 15213 USA; 4 Department of Physical Medicine and Rehabilitation, University of Pittsburgh, Pittsburgh, PA 15213 USA; 5 Department of Veterans Affairs, Pittsburgh, PA 15206 USA; 6 McGowan Institute for Regenerative Medicine, University of Pittsburgh, Pittsburgh, PA 15219 USA

**Keywords:** Electromyography - EMG, Neuromuscular junction, Neurophysiology

## Abstract

Wrist posture impacts the muscle lengths and moment arms of the extrinsic finger muscles that cross the wrist. As a result, the electromyographic (EMG) activity associated with digit movement at different wrist postures must also change. We sought to quantify the posture-dependence of extrinsic finger muscle activity using bipolar fine-wire electrodes inserted into the extrinsic finger muscles of able-bodied subjects during unrestricted wrist and finger movements across the entire range of motion. EMG activity of all the recorded finger muscles were significantly different (p < 0.05, ANOVA) when performing the same digit movement in five different wrist postures. Depending on the wrist posture, EMG activity changed by up to 70% in individual finger muscles for the same movement, with the highest levels of activity observed in finger extensors when the wrist was extended. Similarly, finger flexors were most active when the wrist was flexed. For the finger flexors, EMG variations with wrist posture were most prominent for index finger muscles, while the EMG activity of all finger extensor muscles were modulated in a similar way across all digits. In addition to comprehensively quantifying the effect of wrist posture on extrinsic finger EMG activity in able-bodied subjects, these results may contribute to designing control algorithms for myoelectric prosthetic hands in the future.

## Introduction

The human hand is a complex biomechanical system and both active and passive forces generated from muscles in the forearm are used to maintain postures and perform movements. The movement of a single finger alone generates torque at the wrist^1^ that must be counteracted to maintain wrist posture. Similarly, single digit motion also generates torque in the joints of neighboring digits^2,3^, a phenomenon known as force enslavement, which must in turn be counteracted by other finger muscles. This force enslavement effect has been suggested to be a combination of passive mechanical coupling driven by tendinous connections between digits as well as active coupling from higher order motor commands^2,4^. While there has been significant effort to characterize the resulting electromyography (EMG) activity in the forearm muscles in these scenarios, prior work has primarily used surface EMG recordings and/or examined only a single muscle at a time^5–8^, leaving an incomplete understanding of how the many extrinsic finger muscles are coordinated to produce single and multiple digit movements. Intramuscular EMG is an alternative method of recording EMG signals by placing fine-wire electrodes through the skin directly into muscles. Intramuscular recordings have been used for over 50 years to characterize muscle activity^9–11^ and provides focal recording from a single target muscle that is difficult or impossible to achieve with standard surface EMG. Fine-wire electrodes can also record distinct signals from deep muscles and the different compartments of the multicompartment finger flexor and extensor muscles^12–15^.

Extensive work has demonstrated that coupling between the digits is composed of mechanical^16,17^ and neural components^2,14,15,18,19^, although the influence of inter-digit coupling on the coordination of single and multiple degrees of freedom (DOF) movements is not well understood. Experiments in humans have shown that electrical stimulation induces more focal and independent finger forces compared to volitional movement, suggesting that neural coupling does in fact result from cortical motor control strategies^14^. The influence of coupling is also visible during EMG recordings; an example may be seen in the work by Leijnse *et al.*^5^, which shows phasic EMG activity in the extensor digiti minimi during a thumb tapping task. However, EMG activity of other muscles which appear to be functionally unrelated to the actuation of a joint may contain unique information that is relevant for prosthetic control. Improved EMG decoder performance was observed by Smith *et al.*^20^ when all recorded extrinsic finger muscles were included in the decoder regression equations for each DOF. Conversely, control prosthetic control schemes that rely on muscles crossing just a single joint, such as parallel dual-site control^21^, could lead to inappropriate movements resulting from inter-digit coupling.

Similarly, wrist posture is known to have mechanical effects on the fingers, the most direct being motion of the relaxed fingers while the wrist is flexed and extended. For example, when the wrist is extended the fingers naturally curl into a tenodesis grasp^22^. This occurs because the extrinsic finger flexor muscles span the wrist and are subject to lengthening based on wrist angle, which in turn causes an increase in passive tension in the flexor muscles^23,24^. The resulting increase in force has also been shown to affect the EMG activity necessary to maintain the static pose of a digit^25^. These posture-dependent changes in extrinsic finger flexor EMG have been well-studied in the context of grip force^25–30^ and lead to changes in EMG activity^27,31,32^. Duque *et al.*^31^ quantified this relationship between grip force and EMG as a series of nonlinear models for flexed, extended, and neutral wrist postures, demonstrating that these posture changes alter the relationship between grip force generation and EMG. However, most of these studies have focused on monitoring EMG activity at different wrist postures during tasks where the digits had to exert external loads, such as grasping tasks.

A further motivation to study the relationship between extrinsic finger muscle EMG activity and wrist posture is the potential impact on the design of control systems for prosthetic hands. Dexterous prosthetic hands exist, but a significant barrier to adoption is a lack of control algorithms which take advantage of the high degree-of-freedom movements that are offered in the prostheses^33^. Biomimetically-inspired control algorithms could possibly improve control^34,35^, thus we sought to improve the understanding of the relationship between extrinsic finger muscle EMG activity and wrist posture.

In this study we used intramuscular EMG electrodes to target the compartments of the flexor digitorum profundus (FDP), flexor digitorum superficialis (FDS) and extensor digitorum (ED), as well as the extensor digiti minimi (EDM) and the extensor indicis proprius (EIP) muscle, and sought to characterize the effect of changing wrist joint angles on EMG activity of the extrinsic finger muscles during structured hand movements. We also examined how the extrinsic finger muscles may contribute to wrist movements themselves.

## Methods

### Experimental summary

11 healthy, able-bodied subjects (7 males, 4 females) were included in this study. Subject ages ranged from 25–32 with an average age of 28.2 ± 2.7 years. All subjects provided informed consent prior to any experimental procedure and all procedures were approved by the University of Pittsburgh and Army Research Labs Institutional Review Boards. All methods were performed in accordance with the relevant guidelines and regulations. Sixteen percutaneous EMG electrodes were inserted into the forearm muscles that control finger and wrist flexion and extension movements. Subjects wore a glove fitted with electromagnetic trackers to capture hand kinematics. Subjects then performed an extensive series of single joint movements with varying finger and wrist postures while EMG and kinematics were recorded.

### Percutaneous EMG recording

Percutaneous bipolar fine-wire electrodes^36^ (Motion Lab Systems, Inc., Baton Rouge, LA) were inserted into sixteen forearm muscles. Each electrode pair consisted of two 0.051 mm diameter insulated stainless steel wires. Each wire was passed through a 27 gauge (30 mm long, for superficial muscles) or 25 gauge (50 mm long, for deep muscles) hypodermic needle and the ends of each wire were bent to form a hook. On each wire, 2 mm of insulation was removed from the end to form the active recording area of the electrode, and the two electrodes were offset from each other so that the deinsulated portions did not touch. Each bipolar pair came individually packaged and sterilized and was placed into the target muscle with a single needle insertion. Muscle locations were identified using palpation and ultrasonographic visualization techniques during instructed movements to guide electrode insertion^13^. More specifically, the electrodes were placed by an experienced physician and subjects were asked to make many different movements during the ultrasound procedure and during electrode insertion itself to localize the electrode to the correct muscle or muscle compartment. After the electrode tips were visualized to be in the correct location using ultrasound, the needle was removed, leaving the wires in place as a result of the hooked ends. Care was taken to prevent the wires from becoming dislodged. The subject was asked to exercise the hand and wrist so that the wires moved as deep as possible into the tissue and then a small adhesive bandage was placed at the wire exit site to prevent the wires from being dislodged. After all the electrodes were placed, the arm was wrapped in a bandage to protect the electrodes, lead wires and cabling on the arm for the duration of the experiment.

As this was part of a larger study, some of the implanted electrodes targeted the extrinsic finger muscles while others targeted wrist muscles. In each of the 11 subjects, we recorded from 4–8 extrinsic finger muscles consisting of EIP (n = 6 subjects), ED2 (n = 7 subjects), ED4 (n = 10 subjects), EDM (n = 4 subjects), FDS2 (n = 3 subjects), FDS3 (n = 6 subjects), FDS4 (n = 7 subjects), FDP2 (n = 3 subjects), FDP3 (n = 5 subjects), FDP4 (n = 2 subjects), and FDP5 (n = 4 subjects). Table 1 provides a list of which muscles were recorded in each subject. Intramuscular EMG recordings were digitized with a multichannel neural recording system (Grapevine Neural Interface System with Surf S2 headstage, Ripple, Inc) at 30 kHz.

**Table 1:** Location of electrode placement by subject.

Muscle	Subject											Total per muscle
	1	2	3	4	5	6	7	8	9	10	11	
EIP		X	X	X				X	X		X	6
ED2	X		X	X		X		X		X	X	7
ED4	X	X	X		X	X	X	X	X	X	X	10
EDM					X		X	X		X		4
FDS2	X			X							X	3
FDS3			X	X		X		X	X		X	6
FDS4	X	X			X	X	X	X		X		7
FDP2				X				X		X		3
FDP3			X			X	X		X		X	5
FDP4				X							X	2
FDP5		X	X		X			X				4
Total per subject	4	4	6	6	4	5	4	8	4	5	7	57

### Kinematic motion tracking

**Figure 1:**
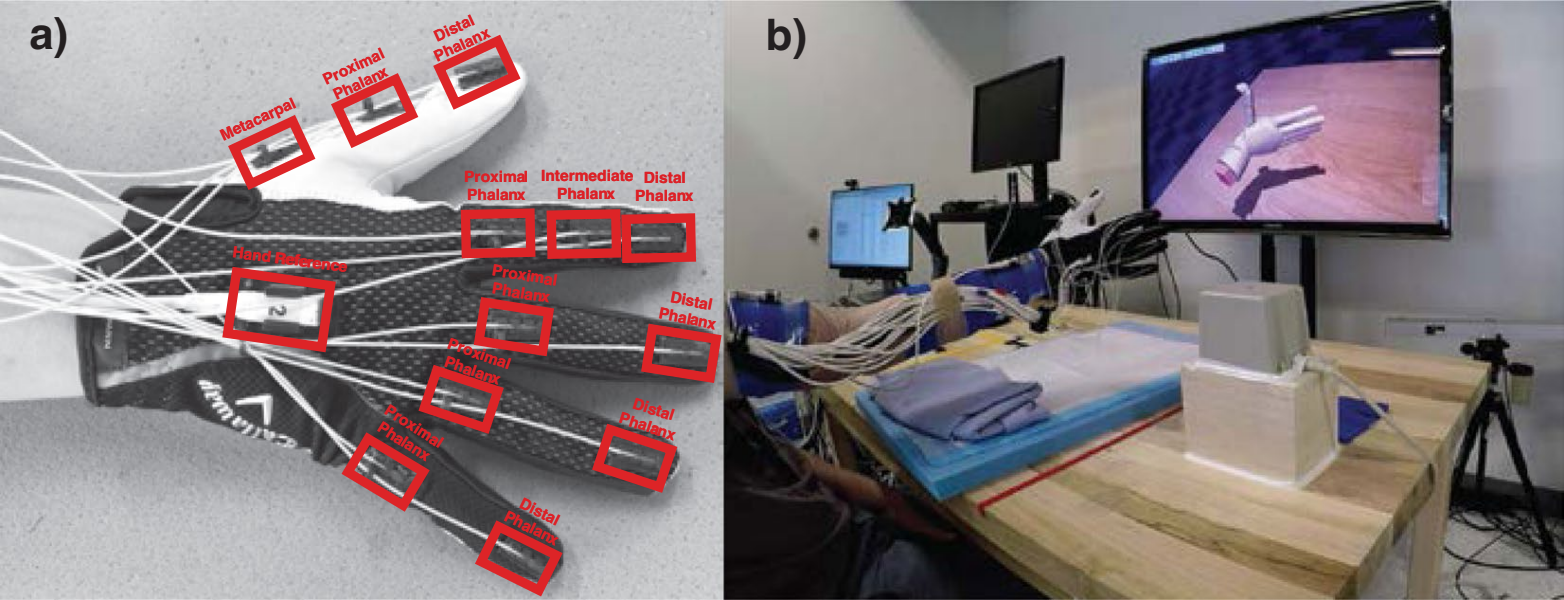
Overview of the experimental setup. (**a**) Electromagnetic tracking glove that subjects wore during the experiments. The red rectangles show placement of electromagnetic sensors in relation to the joints of the fingers. (**b**) Subjects viewed and followed a video which demonstrated the hand posture, movement, and timing.

Hand and arm kinematics were recorded using an electromagnetic tracking system (trakStar, Ascension Technology, Inc.) integrated into the MotionMonitor (Innovative Sports Training Inc., http://www.TheMotionMonitor.com) recording software. The tracking sensors were attached to a glove, and located over the proximal, intermediate, and distal phalanges of the index finger; the proximal and distal phalanges of the middle, ring, and little finger; the metacarpal and phalanges of the thumb; and the dorsal center of metacarpals (Fig. 1a). Tracking sensors were also placed over the distal portion of the radius and lateral aspect of the biceps to track the position of the arm. Subject-specific arm and hand segments were then created using the digitization process in the MotionMonitor software. The OpenSim v3.3 Inverse Kinematics tool^37^ was then used to extract the joint angles from the .trc files using a scaled musculoskeletal model^38^.

### Experimental tasks

Subjects were asked to follow the movements of a virtual hand displayed on a computer screen. Subjects were seated, with their hand and arm held in front of them above the table (Fig. 1b**)**. Each movement exercised a single DOF of the fingers or wrist for ten repetitions at 1 Hz. D2-D5 flexion/extension and wrist flexion/extension movements were tested. A list of all 42 trial types is provided in Supplementary Table S1. Electrodes were never replaced during the experiment, and we saw no evidence that electrode recordings degraded over time. The trials described here were typically completed in 30 minutes or less.

For D2-D5 movements, subjects were asked to produce coupled motions of the metacarpal-phalangeal joint along with the proximal and distal interphalangeal joints, with a focus on moving the metacarpal-phalangeal joint through 90 degrees. All fingers motions were repeated while the subject maintained the wrist in a neutral posture (hand aligned with the forearm and thumb pointing up), or with the wrist in a flexed, extended, pronated, or supinated position at the limit of the wrist range of motion. During wrist movements, the fingers were held in either flexed or extended positions and the exercises were performed while the wrist was held neutral, pronated, or supinated. Pronation/supination movements were only performed during neutral wrist postures. The initial posture for D2-D5 movements was with all fingers extended and the wrist in one of five positions. For wrist movements, the initial starting pose was with the wrist held neutral and the fingers either flexed or extended, depending on the trial.

### Data processing

**Figure 2:**
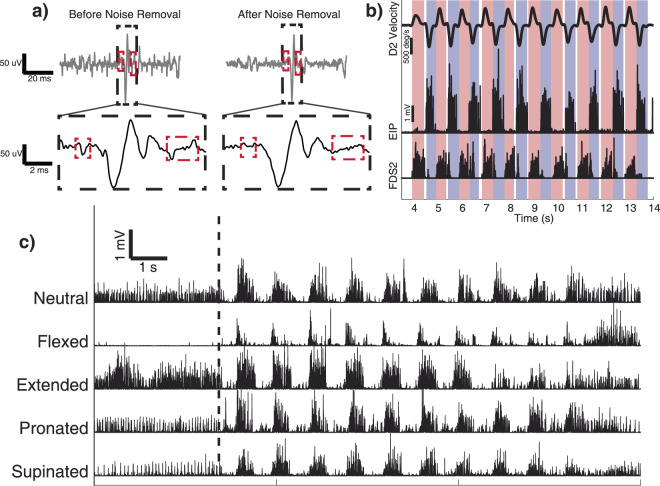
Overview of data processing and effects of wrist posture on EMG activity. (**a**) Comparison of EMG activity during rest before (left) and after (right) electromagnetic noise removal. Blocked in red are examples of electromagnetic artifacts introduced by the kinematic tracking system and the same signal after electromagnetic artifact removal. Left: EMG activity after high-pass filtering to remove DC offset and motion artifacts. The electromagnetic artifacts can be observed boxed in red. Right: The same EMG activity after electromagnetic noise removal. (**b**) D2 metacarpal-phalangeal joint velocity and EMG activity of EIP and FDS2 during repeated flexion (red) and extension (blue) movements. The unshaded region between movements represents brief holding periods that were not included in calculations of EMG activity. (**c**) Rectified and processed EMG activity of ED4 during repetitions of D4 flexion and extension in neutral, flexed, extended, pronated, and supinated postures. The dashed vertical line shows the task start cue. The hand was held in these static postures for approximately 4 seconds before performing 10 movement repetitions. Note the substantial change in EMG activity for the same finger movement in different wrist postures.

Kinematic and EMG signals were processed offline using Matlab (The Mathworks, version 2016b). Kinematic data were smoothed with a 4^th^ order low-pass Butterworth filter at 10 Hz to remove noise from the motion tracking system. The single-ended intramuscular EMG recordings were first differenced and the resulting signals were then high-pass filtered with a 4th order high-pass Butterworth filter at 10 Hz in order to remove low-frequency drift in the signal. Next, each signal underwent a process to remove electromagnetic noise generated by the kinematic motion tracking system (Fig. 2a**)**. The electromagnetic field was captured by the EMG recording system as a series of 100 µs pulses occurring at the pulse frequency of 100 Hz. We removed these narrow pulses from recordings offline by generating a template pulse train, and then subtracting it from the data. An example of the EMG signal before and after electromagnetic noise removal is shown in Fig. 2a. After electromagnetic noise was removed, EMG signals were band-pass filtered with a 4^th^ order Butterworth for 100 to 4000 Hz to remove movement artifacts, line noise, and high frequency noise. Similar bandpass filter settings are commonly used in intramuscular EMG recordings^39–41^.

Potential extrinsic finger muscle recordings were excluded during the data analysis phase if the EMG activity from an electrode was not modulated by single digit movement^42,43^, or if the electrode recordings were significantly contaminated by artifacts which occurred occasionally with the fine-wire electrodes. Artifacts were identified based on signal amplitude and shape. Events with peak voltages much greater than those occurring during a maximal voluntary contraction, or events with large instantaneous changes in amplitude or that did not resemble typical EMG activity or motor unit action potentials were considered artifacts. If multiple electrodes were in the same muscle or muscle compartment for a subject, we included only a single electrode with the highest signal-to-noise ratio. Electrode placement was verified after experiments by reviewing EMG activity from D1-D5 flexion/extension movements in neutral, flexed, extended, supinated, and pronated postures as well as from 14 trials of wrist movements.

### Data analysis and statistics

We divided the EMG data for every trial into flexion and extension phases using the kinematic recordings (Fig. 2b). The angular velocities were low-pass filtered with a zero-phase digital filter at 10 Hz. We detected the movement phases by first identifying the positive and negative peak velocities. Movement onset was then detected based on the first zero velocity point preceding each peak, while the end of a movement was the first zero velocity point after a peak. The filtered EMG data were integrated over the relevant movement windows and divided by the duration of the movement to quantify the average EMG activity over the period. For example, the flexor digitorum superficialis was integrated during flexion movements and the extensor digitorum was integrated during extension movements.

#### EMG normalization

Average EMG activity was normalized separately for finger and wrist movement analyses. As the primary objective of these analyses was to compare the way that EMG activity changed across different postures, we choose to normalize activity across the trials themselves, rather than to a maximal voluntary contraction. For finger analyses, mean EMG activity was calculated for each muscle and subject during digit movements in all wrist postures (e.g. Fig. 2c). The EMG activity for each muscle was then normalized to the mean value from the wrist posture that evoked the largest EMG activity. For example, in Fig. 2c, the average EMG activity of ED4 was highest when the wrist was extended. Therefore, all ED4 EMG activity for this subject was normalized to the mean value that occurred while the wrist was extended. Similarly, for wrist analyses, the mean EMG activity for each finger muscle was calculated for the four combinations of finger postures and wrist movements: fingers extended (‘flat’) during wrist flexion and extension and fingers flexed (‘fist’) during wrist flexion and extension. For the four movements, the EMG activity of each muscle and subject was normalized to the mean peak activity during the task.

#### Finger movements

The normalized EMG activity of each muscle and compartment were tested for significant differences using a two-way ANOVA with subject and wrist posture (neutral, flexed, extended, pronated, and supinated) as factors. Post-hoc pairwise testing to identify differences in EMG activity for movements in the postures assessed was performed using Tukey’s honest significant differences (HSD) test for multiple comparisons.

#### Wrist movements

The normalized EMG activity of ED, EDM and EIP muscles were combined into a single ‘extensors’ group, while FDS and FDP were combined into a ‘flexors’ group. Since the data were not normally distributed (p < 0.05, Kolmogorov-Smirnov test,) a Kruskal-Wallis test was conducted on the EMG activity of the extrinsic finger flexors and extensors to detect whether EMG activity of finger flexors and extensors differed based on finger position. Post-hoc pairwise testing was performed using Dunn’s test with a Bonferroni correction.

## Results

### Finger movements at different wrist postures

#### Finger extensors

**Figure 3:**
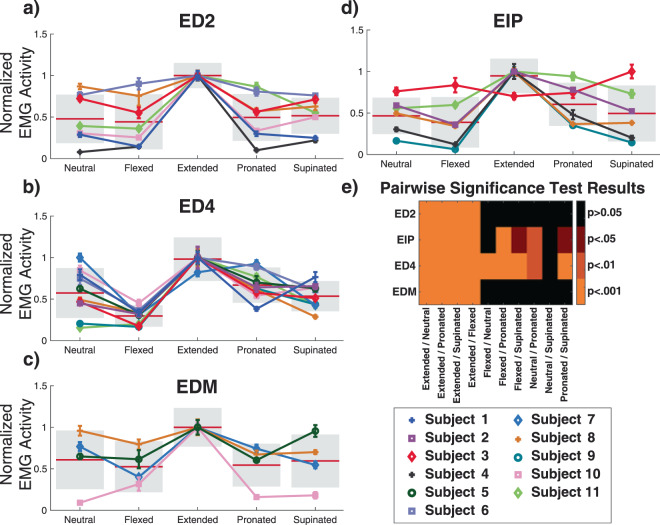
(**a–d**) Overall mean normalized EMG activity for each subject for the extrinsic finger extensors during extension motions with the wrist in neutral, flexed, extended, pronated, and supinated postures. All finger extensors had their maximum EMG activity during extension movements when the wrist was held extended. Individual data points represent the subject mean and error bars are standard error of the mean. Data are color and marker coded for each subject. The horizontal red bars at each wrist posture show normalized group means and the standard deviation across subjects is shown as a gray box. Pairwise comparisons of EMG activity between the wrist extended and all other postures showed significant differences (p < 0.001, Tukey’s HSD). (**e**) Heat map of pairwise significant differences for EMG activity of the extrinsic finger extensor muscle for all combinations of wrist postures.

For all the finger extensor muscles, both posture and subject had a significant main effect on EMG activity (p < 0.0001, ANOVA). Further, the EMG activity of every finger extensor muscle was highest when the wrist was held in an extended posture. This activity was significantly higher than all other postures assessed (p < 0.001, Tukey’s HSD, Fig. 3**)**. In contrast, when the wrist was flexed, the finger extensors generated the lowest level of EMG activity during the extension phase of the motion, ranging from mean levels of 30 ± 13% to 53 ± 30% of their maximum activity level when the wrist was extended. These significant changes in EMG activity in response to wrist posture were consistent across all of the extensor muscles with the highest levels of EMG activity occurring when the wrist was extended and the lowest levels occurring when the wrist was flexed. EMG activity for the finger extensors with the wrist in the neutral, pronated, and supinated postures was at an intermediate level and varied depending on the muscle. When the wrist was held neutral during digit extension movements, average EMG activity ranged from 47 ± 22% to 61 ± 35% of its maximum value with the lowest activity occurring in the D2 extensors (ED2, EIP) and progressively increasing for ED4 and EDM. Average EMG activity when the wrist was pronated ranged from 50 ± 28% to 67 ± 21%. Notably, the two index finger extensor muscles were differentially modulated when the wrist was pronated. While ED2 and EIP activity were similar when the wrist was in the neutral, extended or supinated postures, the average EMG activity for ED2 and EIP was 50 ± 28% and 61 ± 26%, respectively when the wrist was pronated. This effect may be driven by the differing origin and insertion points of the ED and EIP muscles, which are accentuated during wrist pronation. With the wrist supinated, EMG activity across all the finger extensors varied the least, with all muscles having average activity levels between 49 ± 33% and 59 ± 31% of their maximum.

Of the extensor muscles assessed, ED4 showed the greatest difference in activity based on wrist posture. Of the ten possible pairwise comparisons (e.g. neutral vs. flexed, neutral vs. extended), all but neutral vs. supinated postures showed significant differences (p < 0.01, Tukey’s HSD). ED2 and EDM were affected the least by changing wrist posture; only pairwise comparisons testing extension against the other postures showed statistical significance (Fig. 3e**)**.

#### Finger flexors

**Figure 4:**
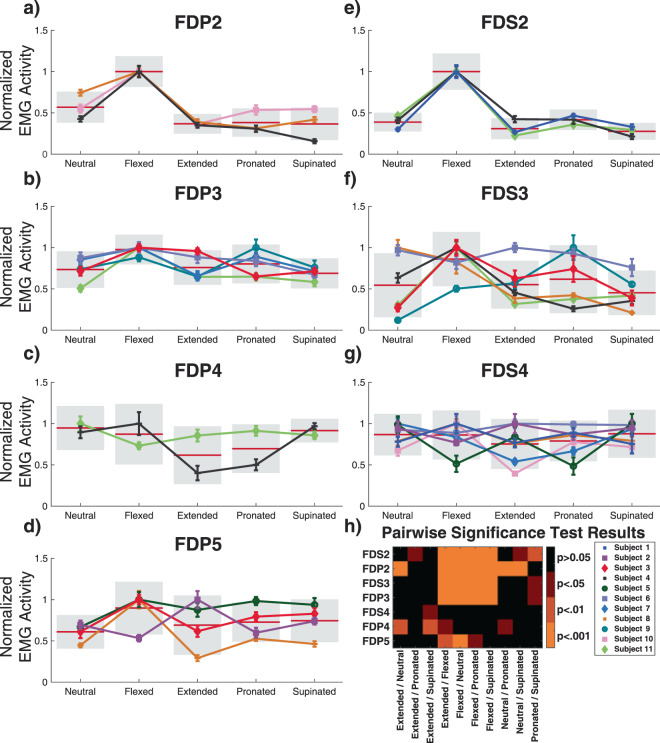
(**a–g**) Overall mean normalized EMG activity for each subject for the extrinsic finger flexors during flexion motions with the wrist in neutral, flexed, extended, pronated, and supinated postures. The D2, D3, and D5 finger flexors showed significantly elevated EMG activity when the wrist was flexed. Individual data points represent the subject mean and standard error of the mean. Data are color and marker coded for each subject. The horizontal red bars show normalized group means and the standard deviation is shown as a gray box. (**h**) Heat map of pairwise significant differences (Tukey’s HSD) of EMG activity between postures for the extrinsic finger flexors.

Similar to the finger extensors, wrist posture had a significant effect on the EMG activity of all of the finger flexor muscles (p < 0.001 for all muscles except FDS4, p < 0.05, ANOVA with Tukey’s HSD). The finger flexor muscles were most active when the wrist was held in the flexed posture, with the exception of FDS4 and FDP4. FPD4 was most active in a neutral posture with an average normalized EMG activity of 95 ± 26%, although the low number of muscle recordings could have an effect on this result. FDS4 was also most active in the neutral wrist posture with an average normalized EMG activity of 87 ± 25%, although this was not statistically higher than other wrist postures. With the wrist in the neutral posture, the other flexor muscles had average activity levels that were much lower and ranged between 54 ± 18% to 73 ± 22% (Fig. 4**)**. When the wrist was extended, the EMG activity of the flexor muscles required to produce the same finger flexion motion ranged from just 31 ± 12% (FDS2) to 76 ± 21% (FDP3) of that required when the wrist was flexed. In contrast to the finger extensors, the individual finger flexor muscles showed little difference between extended, pronated, and supinated postures. Interestingly, the overall effect of wrist posture on EMG activity was more prevalent for D2 and D3 compared to D4 and D5 (Fig. 4), whereas all finger extensors were affected similarly by changes in wrist posture (Fig. 3).

#### Inter-subject variance

For all muscles that were assessed, excluding FDS2, the individual subject had a significant effect on how wrist posture effected extrinsic finger muscle EMG activity (p < 0.05, Tukey’s HSD). This main effect response was primarily driven by high responders, which were subjects that had particularly high levels of finger EMG activity when the wrist posture matched the action of the finger muscle (e.g. wrist extension for finger extensors). ED2 in Subjects 1 and 4 (Fig. 3a**)**, EDM in Subject 10 (Fig. 3c**)**, EIP in Subjects 4 and 9 (Fig. 3d) and FDP5 in Subject 8 (Fig. 4d**)** are all examples of this. Overall this effect was less pronounced in the finger flexor muscles. There were a few other notable instances of inter-subject variance that we observed in these data. For example, the FDP5 EMG activity in Subject 2 (Fig. 4d) was maximized when the wrist was extended, rather than flexed, as was the case for all other subjects. In this case, the EMG activity clearly showed that this electrode was in the FDP5 muscle as it highly active only during flexion of the 5^th^ digit. In this subject, motion of the 5^th^ digit was atypical in that most of the flexion and extension occurred at the proximal interphalangeal joint when the wrist was extended, rather than the metacarpal-phalangeal joint.

### Extrinsic muscle finger EMG activity during wrist movements

**Figure 5:**
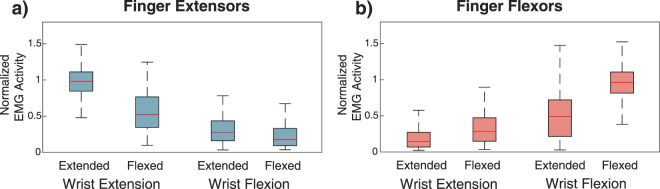
Normalized mean EMG activity for (**a**) finger extensors (N = 22 muscles) and (**b**) finger flexors (N = 28 muscles) during wrist extension and flexion movements in flat and fist postures. For each of the 11 subjects, 10 repetitions were performed for movements in each posture. The red line shows the median, and the outer boxes are the first and third quartile. Error bars represent the 5–95% confidence interval. All posture and movement combination showed significant differences (p < 0.005, Kruskal-Wallis with Dunn post-hoc testing).

The extrinsic finger extensor muscles were highly active during wrist extension regardless of the finger posture. In fact, finger extensor EMG activity was significantly higher when the fingers were fully flexed and the wrist was being extended than when the fingers were extended and the wrist was being flexed (p < 0.001, Dunn’s, Fig. 5a). In the four combinations assessed (wrist extension/flexion with fingers extended/flexed), the extrinsic finger extensor EMG showed a wide range of activity levels. The highest EMG activity occurred during wrist extension when the fingers were held extended where the median normalized EMG activity from the finger extensors was 98% (IQR 85%–111%). When the wrist was extending and the fingers were held flexed, the median normalized EMG for the finger extensors declined to 52% (IQR 35%–77%). During wrist flexion movements, finger extensor EMG activity was 28% (IQR 16%–44%) when the fingers were held extended and was even smaller at 18% (IQR 9%–33%) when the fingers were held flexed. All pairwise comparisons of EMG activity for the different movement and posture combinations were significantly different (p < 0.001, Kruskal-Wallis test with Dunn’s post-hoc test). Overall, we found that the finger extensor EMG activity changed by more than a factor of three when the fingers were held extended based on whether the wrist was moving in extension or flexion.

The EMG activation patterns of the finger flexor muscles showed a similar pattern to that observed in the finger extensors, with finger flexors significantly more active during wrist flexion movements than wrist extension movements, even when wrist extension was performed with the fingers in a fist (Fig. 5b**)**. The highest EMG activity of the movements and postures assessed occurred during wrist flexion when the fingers were fully flexed at a median normalized EMG activity of 96% (IQR 82%–111%). The next highest level of EMG activity was during wrist flexion with the fingers extended with a median normalized value of 49% (IQR 21%–72%), nearly half of the highest value. Even when the fingers were being actively held in extension, finger flexor muscles were active during wrist flexion movements. EMG activity for the finger flexors was lowest during wrist extension movements; during extension movements with the fingers flexed, the median normalized EMG activity was 29% (IQR 15%–47%). When the wrist was extended and the fingers were extended, median normalized EMG activity was the lowest at 15% (IQR 7%–27%). The difference in activity levels were all significant (p < 0.001, Kruskal-Wallis test with Dunn’s post-hoc test) and varied by more than threefold depending on whether the wrist was flexing or extending when the fingers were fully flexed.

## Discussion

In this study, we found that there were wrist posture-dependent effects on extrinsic finger muscle EMG activity, which is consistent with previous reports^8,31,32,44^. We also expanded on this work by investigating EMG activity from the individual muscle compartments of the FDS, FDP and ED muscles, specifically while using completely unloaded finger movements like would occur during hand pre-shaping in reach-to-grasp actions^45,46^. An important issue is that using intramuscular electrodes instead of surface electrodes increased the likelihood that the recorded signals were indeed from the same muscle in all postures. With surface EMG, there can be significant motion of the muscles under the skin, making it difficult to ensure that recordings at all wrist postures would be from the same muscle^8,31^.

In these experiments, we found that during single digit motions, both the finger flexors and extensors exhibited significant changes in their EMG activity based on the posture of the wrist, and in some cases varied by as much as 70% of their normalized value. We have also shown evidence that the extrinsic finger muscles have higher levels of activity during wrist movements that are performed in the same direction of action regardless of finger posture, which suggests that these muscles assist with unloaded wrist movements. These muscles may therefore also play a role in maintaining static wrist postures, which is consistent with other studies^27^.

There are multiple causes for EMG activity to change based on wrist posture. Previous work has shown that grip and fingertip forces decrease as wrist posture moves further into a flexed posture^29,30^. It has been suggested that this may be in part due to the diminished force production capacity of the flexors as they shorten^30^. If the required force output or tasks remains the same and force production ability diminishes, an increase in EMG activity is required to increase force generation^47,48^.

Posture dependent activation levels may also be attributed to the altered tension of the antagonist extrinsic finger muscles caused by lengthening of muscles and tendons during different wrist postures, which change the force necessary to make such movements^49^. The increased passive force of the extrinsic finger muscles when the is wrist extended and flexed has been previously documented^23,30,50^. For example, when the wrist is extended, the FDS and FDP muscles are lengthened, which generates an increased flexion torque on the digits^51,52^. The extensor digitorum and other assisting muscles must therefore generate more force, and thus EMG activity, to counteract the increased tensions of the flexor muscles both to maintain finger position and to perform extension movements. During finger flexion movements while the wrist is still held extended, the lengthened finger flexor muscles generate substantial passive forces that assist in flexion movements and therefore require less EMG activity to make a flexion movement. This may also explain why the flexor compartments of the radial digits (FDS2, FDS3, FDP3) had the lowest activation in the supinated posture, while the flexor compartments of the ulnar digits (FDS4, FDP4, FDP5) were substantially more active in the same posture. Despite the muscle compartments showing consistent patterns of responses, such as elevated EMG activity of the finger extensors when the wrist was extended and lower activity when the wrist was flexed (Fig. 3), there were differences in the magnitude of those responses across fingers. This may be explained in part by the differences in passive forces generated by the individual compartments at different wrist postures. The study by Keir *et al.*^23^ showed that passive muscle force differences of up to 200% may occur between compartments of the same muscle, such as in the case of FDS2 and FDS4. The moment contributions of FDS and FDP also vary across the digits^52^. It is therefore plausible that the extrinsic finger muscle compartments exhibit some differences in response magnitude based on wrist posture, but that the overall pattern of responses is maintained.

EMG activity was subject-dependent and the effect of the subject was found to be significant for nearly all muscles. This is due in part to instances where a muscle was primarily active in a single wrist posture, and much less active, in some cases just 10–30% of the normalized activity, in other wrist postures (e.g. Fig. 3a,d). Interestingly, the EMG activity patterns of these high responders appeared to be limited to a single muscle. For example, EDM of subject 10 showed high EMG activity during the extended posture but low activity during the other postures (Fig. 3c**)**. In the other muscles and movements of subject 10, the EMG activity showed a range of responses similar to other subjects (Figs. 3a,b and 4a,f**)**. Although high responders had less pairwise differences in EMG activity due to posture, their performance still demonstrates that EMG activity for movements is affected by wrist posture. It is unsurprising that differences between subjects were observed. Control over the hand and fingers has been shown to vary substantially across subjects^53,54^ and the number of muscles within the hand lends itself to redundant control where different muscle activity combinations could be used to achieve similar kinematic outputs^55^.

A core limitation of our work was that we were unable to determine how much or the change in extrinsic finger EMG activity based on posture was due to the necessity of overcoming mechanical coupling effects, such as the changing passive forces of antagonist muscles lengthening, as opposed to a motor control strategy in which the fingers assist in maintaining wrist posture. We are therefore unable to directly ascribe whether this phenomenon is a reaction to passive forces or a method of control that uses multiple muscles for force generation. Electrode placement was also a challenge in certain cases. For example, we didn’t have electrodes in ED3 due in part to the location of the posterior antebrachial cutaneous nerve. Electrode placement in FDS4 and FDP4 also proved challenging. Four electrodes that were targeted to FDP4 penetrated too deeply and were believed to be located in the flexor carpi ulnaris muscle in post hoc experiment data analysis, as the electrodes showed no response to finger movement but were active during wrist flexion and adduction. We were unable to determine whether this was due to the small size of these muscles or electrode migration that may have occurred immediately after placement while subjects first moved their hands. Another limitation on our work was the testing of pronation and supination postures. While we did test finger movements in these wrist postures, we did not explore activity during pronation and supination movements as thoroughly as Mogk *et al.*^32^ and it was difficult to draw meaningful conclusions on these postures, as there did not seem to be a consistent behavior across the muscles examined. Biomechanical models of the arm and hand may assist in understanding these postures, as the EMG activity of the extrinsic finger muscles may be impacted by the changing muscle lengths and moment arms which can be modeled in simulation software.

In this study we demonstrated that wrist posture significantly influences the necessary action of finger muscles as demonstrated by the large changes in EMG activity of the extrinsic finger muscles in able-bodied subjects. Indeed, in certain cases, a three-fold change in finger muscle activity was required to produce the same finger kinematics when the wrist was held in different postures. For the finger flexors, these effects were most pronounced for muscles controlling the index finger. Future work could investigate whether the effects of wrist posture on finger muscle activity are still present in traumatic transradial amputees where these mechanical influences are removed. This could help clarify whether these interactions represent a learned feed-forward control strategy driven by the cortex, or might be predominantly driven by real-time sensory-driven feedback.

## Supplementary information

Supplementary Table 1.

## Data Availability

The dataset is available from the corresponding author on reasonable request.
