# Supplementary material for: The effect of wrist posture on extrinsic finger muscle activity during single joint movements
Source: Sci Rep. 2020 May 20;10:8377. doi: 10.1038/s41598-020-65167-x (PMC7239904; doi:10.1038/s41598-020-65167-x)
Supplement: Supplementary file 1 — Supplementary Table 1. [file 41598_2020_65167_MOESM1_ESM.pdf]

The effect of wrist posture on extrinsic finger muscle activity during single joint movements

Carl R. Beringer*[1,2,3], Misagh Mansouri[1,4], Lee E. Fisher[1,2,3,4], Jennifer L. Collinger[1,2,3,4,5], Michael C. Munin[4], Michael L. Boninger[1,2,4,5,6], Robert A. Gaunt*[1,2,3,4]

[1]Rehab Neural Engineering Labs, University of Pittsburgh, Pittsburgh, PA 15213, USA
[2]Department of Bioengineering, University of Pittsburgh, Pittsburgh, PA 15213, USA
[3]Center for the Neural Basis of Cognition, Pittsburgh, PA 15213, USA
[4]Department of Physical Medicine and Rehabilitation, University of Pittsburgh, PA 15213
[5]Department of Veterans Affairs, Pittsburgh, PA 15206, USA
[6]McGowan Institute for Regenerative Medicine, University of Pittsburgh, Pittsburgh, PA 15219, USA

Correspondence to: Robert Gaunt rag53@pitt.edu

Supplementary Tables

Supplementary Table 1: List of movements performed for each subject. For trial types 1-30 the wrist posture column indicates the static wrist posture for the entire trial, while the finger posture column indicates the position of the other fingers during the movement. For trial types 31-42 the wrist posture column indicates the wrist rotation posture for the entire trial, while the finger posture column indicates the position of all the fingers during the movement.

Trial #	Movement	Wrist Posture	Finger Posture
1	D2 Flexion/Extension	Neutral	Extended
2	D3 Flexion/Extension	Neutral	Extended
3	D4 Flexion/Extension	Neutral	Extended
4	D5 Flexion/Extension	Neutral	Extended
5	D1 Flexion/Extension	Neutral	Extended
6	D1 Abduction/Adduction	Neutral	Extended
7	D2 Flexion/Extension	Flexed	Extended
8	D3 Flexion/Extension	Flexed	Extended
9	D4 Flexion/Extension	Flexed	Extended
10	D5 Flexion/Extension	Flexed	Extended
11	D1 Flexion/Extension	Flexed	Extended
12	D1 Abduction/Adduction	Flexed	Extended
13	D2 Flexion/Extension	Extended	Extended
14	D3 Flexion/Extension	Extended	Extended
15	D4 Flexion/Extension	Extended	Extended
16	D5 Flexion/Extension	Extended	Extended
17	D1 Flexion/Extension	Extended	Extended
18	D1 Abduction/Adduction	Extended	Extended
19	D2 Flexion/Extension	Pronated	Extended
20	D3 Flexion/Extension	Pronated	Extended
21	D4 Flexion/Extension	Pronated	Extended
22	D5 Flexion/Extension	Pronated	Extended

23	D1 Flexion/Extension	Pronated	Extended
24	D1 Abduction/Adduction	Pronated	Extended
25	D2 Flexion/Extension	Supinated	Extended
26	D3 Flexion/Extension	Supinated	Extended
27	D4 Flexion/Extension	Supinated	Extended
28	D5 Flexion/Extension	Supinated	Extended
29	D1 Flexion/Extension	Supinated	Extended
30	D1 Abduction/Adduction	Supinated	Extended
31	Wrist Flexion/Extension	Neutral	Flexed
32	Wrist Flexion/Extension	Pronated	Flexed
33	Wrist Flexion/Extension	Supinated	Flexed
34	Wrist Flexion/Extension	Neutral	Extended
35	Wrist Flexion/Extension	Pronated	Extended
36	Wrist Flexion/Extension	Supinated	Extended
37	Wrist Abduction/Adduction	Neutral	Flexed
38	Wrist Abduction/Adduction	Pronated	Flexed
39	Wrist Abduction/Adduction	Supinated	Flexed
40	Wrist Abduction/Adduction	Neutral	Extended
41	Wrist Abduction/Adduction	Pronated	Extended
42	Wrist Abduction/Adduction	Supinated	Extended
